# 

**Published:** 1879-04

**Authors:** 


### Books Received.

---

Clinical Lectures on Diseases peculiar to women. By Lombe Atthill, M. D., University Dublin. Philadelphia: Lindsay & Blakiston. Buffalo: T. H. Butler; pp. 335.

On loss of weight, blood-spitting and lung disease. By Horace Dobell, M. D.

Consulting Physician to the Royal Hospital for diseases of the chest, etc. Philadelphia: Lindsay & Blakiston. Buffalo: T. H. Butler; pp, 267.

The National Dispensatory, containing the Natural History, Chemistry, Pharmacy, Actions and uses of Medicines, including those recognized in the Pharmacopœias of the United States and Great Britain. By Alfred Stillé, M. D.; L.L. D. and John Maisch, Ph. D., with two hundred and one illustrations. Philadelphia: Henry C. Lea. Buffalo: T. H. Butler; pp, 1540.

Naval Hygiene, Human Health and the means of preventing disease, with Illustrative Incidents principally derived from naval experience. By Joseph Wilson, M. D., Medical Director U. S. Navy. Second Edition with colored Lithographs, etc. Philadelphia: Lindsay and Blakiston. Buffalo: T. H. Butler, pp. 257.

Lectures on Practical Surgery. By H. H. Toland, M. D., Professor of the Principles and Practice of Surgery and Clinical Surgery in the Medical Department of the University of California. Philadelphia: Lindsay & Blakiston,. Buffalo: T. H. Butler.

Modern Surgical Therapeutics. A Compendium of Current Formulæ, approved dressings and specific methods of Surgical Diseases and Injuries. By George H. Napheys, A. M., M. D., etc. Philadelphia: D. G. Brinton, 115 South Seventh street, pp, 590.

The Diseases of Live Stock, and their most efficient Remedies, including Horses, Cattle, Sheep and Swine. By Lloyd V. Tellor, M. D. Philadelphia: D. G. Brinton, 115, South Seventh street, pp, 458.

Transactions of the American Medical Association, Vol. 29, 1878: Philadelphia.

A Clinical Treatise on Diseases of the Liver. By Dr. Fried. Theod. Frerichs, Professor of Clinical Medical in the University of Berlin, etc; in three volumes. Vol. I, Translated by Charles Murchison, M. D., F. R. C. P., Physician to the London Fever Hospital. New York: Wm. Wood & Co., 27 Great Jones street, pp, 219.

Physical Therapeutics; A New Theory. By Thomas W. Poole, M. D., M. C. P. S., Ont. Toronto: The Toronto News Company. New York: The American News Company. London: The International News Company, pp, 231.

———:o:———

### Pamphlets Received.

Epidemics from a Chemical Standpoint; a lecture delivered by R. Ogden Doremus, M. D., L. L. D., before the Medico-Legal Society of New York.

The First Annual Report of the Presbyterian Eye and Ear Charity Hospital, Baltimore, Md.

Reports of the Trustees and Superintendents of the Butler Hospital for the Insane, Providence, R. I.

Clinical Lecture on Syphilitic Brain Lesions. By Ed. C. Seguin, M. D.

The Diagnosis of Progressive Locomotor Ataxia. By E. C. Seguin, M. D.

Report of the Pennsylvania Hospital for the Insane, for the year 1878.

History of the Medical Society of Oneida Co. from its organization July 1806, to July 1878.

Report of Investigations into the Pathology of Diphtheria, conducted by Edward Curtiss, M. D., and Thomas E. Satterthwaite, M. D., New York.

An Address presenting the Claims of the Medical Department. Read before a "Council" in the interests of Syracuse University. By Alfred Mercer, M. D.

Report on Aconitia in Trigeminal Neuralgia. By E. C. Seguin, M. D.

A Contribution to the Medicinal Treatment of Chronic Trigeminal Neuralgia. By E. Seguin, M. D.

Conclusions of the Board of Experts, authorized by Congress to investigate the Yellow Fever Epidemic of 1878.

An Essay of Malpractice; read before the Luzerne county Medical Society at Wilkes-Barre, January 8th, 1879. By John B. Crawford, M. D.

Twenty-fifth Annual Report of the Managers of the State Lunatic Asylum, Utica, N. Y., for the year 1877.

Disease Germs; their origin, nature, and relation to wounds. By B. A. Watson, M. D., Jersey City, N. J.

A case of Progressive Muscular Atrophy with Sclerosis of the Lateral Columns. By Dr. J. C. Shaw, President of the New York Neurological Society, Medical Director of Kings county Insane Asylum.

The Causes of Sudden Death of Puerperal Women. An Address delivered before the American Medical Association, June 5, 1878. By Edward W. Jenks, M. D., Detroit, Mich.

Nineteenth Annual Report of the Medical Superintendent of the State Asylum for Insane Criminals, Auburn, N. Y., for the year ending September 30, 1878.

Oyster-Shuckers Comeitis, (Comeitis Ostrearii) By W. J. McDowell, M. D. Read before Baltimore Medical and Surgical Society Jan. 16, 1879.

Transactions of the American Dermatological Association, at the Second Annual Meeting held at Saratoga Springs, August, 1878.

On the Fracture of the Femur. By Edward Borck, M. D., St. Louis, Mo.

The Therapeutic value of Ergot. By J. W. Compton, M. D., Professor of Materia Medica and Therapeutics in the Medical College of Evansville, Ind.

The Non-Asylum Treatment of the Insane. By Wm. A. Hammond, M. D.

Malt-Liquors, Their influence on Digestion and Nutrition. By J. J. Coleman, F. I. C., F. C. S.

a9 (4 cop.)

### THE BUFFALO

### MEDICAL AND SURGICAL

MEDICAL AND SURGICAL JOURNAL.

PUBLISHED MONTHLY,—Containing Original Articles, Reports of Medical Societies and Hospitals, Editorial, Reviews, Correspondence, Army News, etc., etc ; including the usual variety of Medical Periodical Publications. Specimen copies sent on application to BUFFALO MEDICAL AND SURGICAL JOURNAL, J. F. MINER, M. D., 978 Main Street, BUFFALO, N. Y.

TERMS OF SUBSCRIPTION, $3 00 PER YEAR, IN ADVANCE,—All communications for publication should be sent before the fifteenth (15) of each month, or they will of necessity lie over until the next number. No change can be made in advertisements after the twenty-fifth.

Correspondence and original articles are solicited. Publication is no evidence that the views of the author are endorsed by the Journal.

